# Epigenetic Biomarkers of Preterm Birth and Its Risk Factors

**DOI:** 10.3390/genes7040015

**Published:** 2016-04-13

**Authors:** Anna K. Knight, Alicia K. Smith

**Affiliations:** 1 Genetics and Molecular Biology Program, Emory University, Atlanta, GA 30322, USA; anna.knight@emory.edu; 2 Department of Psychiatry & Behavioral Sciences, Emory University School of Medicine, Atlanta, GA 30322, USA

**Keywords:** epigenetics, biomarker, preterm birth, methylation, pregnancy, gestation

## Abstract

A biomarker is a biological measure predictive of a normal or pathogenic process or response. Biomarkers are often useful for making clinical decisions and determining treatment course. One area where such biomarkers would be particularly useful is in identifying women at risk for preterm delivery and related pregnancy complications. Neonates born preterm have significant morbidity and mortality, both in the perinatal period and throughout the life course, and identifying women at risk of delivering preterm may allow for targeted interventions to prevent or delay preterm birth (PTB). In addition to identifying those at increased risk for preterm birth, biomarkers may be able to distinguish neonates at particular risk for future complications due to modifiable environmental factors, such as maternal smoking or alcohol use during pregnancy. Currently, there are no such biomarkers available, though candidate gene and epigenome-wide association studies have identified DNA methylation differences associated with PTB, its risk factors and its long-term outcomes. Further biomarker development is crucial to reducing the health burden associated with adverse intrauterine conditions and preterm birth, and the results of recent DNA methylation studies may advance that goal.

## 1. Introduction

The prenatal environment has become increasingly recognized as an important predictor of immediate and long-term risk for chronic conditions, but there are few biological markers that identify those at risk for common pregnancy complications, such as preterm birth (PTB). Early identification of mothers at increased risk for preterm delivery or of children at risk of developmental consequences resulting from preterm birth may facilitate effective prenatal or postnatal interventions. Even in the absence of a fully effective preventative treatment for PTB, measures can be taken to mitigate risk for the mother and fetus. Potential interventions for the mother may include preemptive monitoring and preparation for labor in a facility prepared to care for high-risk women and neonates. Mothers who are more likely to deliver preterm may also benefit from prenatal social support programs. Interventions for the fetus may include the anticipation of clinical interventions to limit complications common in preterm infants as well as early participation in developmental enrichment programs [1]. Over the past several years, the availability of cost-effective methods for assessing the epigenome has led to identification of epigenetic signatures of the intrauterine environment including exposure to medication, stress, gestational length, and smoking [2,3,4,5,6,7,8,9,10,11,12,13]. Evaluation of the genes containing these differentially methylated sites is not only useful for identifying biological pathways regulated in each circumstance, but it may also facilitate identification of clinically informative biomarkers.

A biomarker is a biological measure that is predictive of a normal or pathogenic process or response. In clinical practice, biomarkers can be used for risk assessment, early detection or onset of a disease or chronic illness. Once a diagnosis is established, they can also be used as an indicator of symptom severity or response to treatment [14,15,16]. In general, a biomarker candidate must be reproducible and have sufficient sensitivity and specificity to provide clinically-relevant information [17].

**Figure 1:**
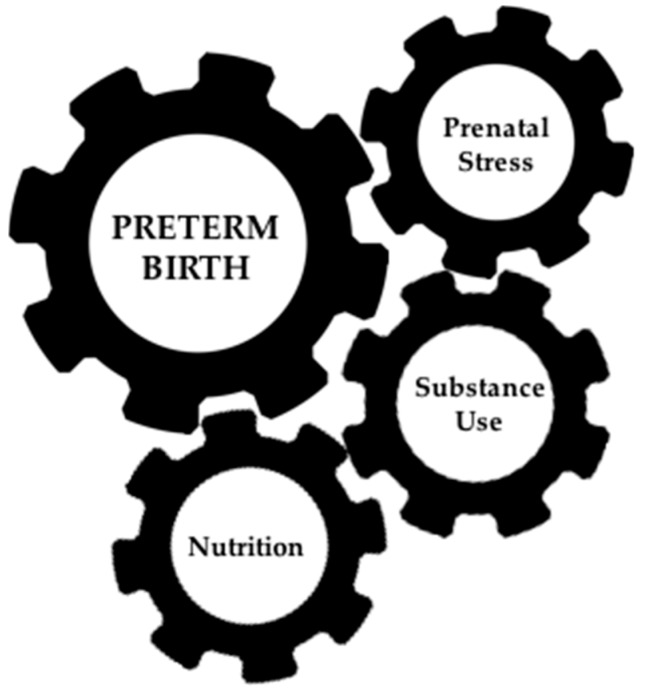
Nutrition, substance use and stress during pregnancy increase risk for spontaneous preterm birth. Each of these risk factors has been associated with DNA methylation differences that may be informative for early prediction and treatment.

Many factors influence the prenatal environment, and this review is not meant to extensively cover all of them. We will focus on recent epigenetic studies of preterm birth and environmental factors that increase maternal risk for PTB and infant risk for chronic conditions across the lifespan including maternal perinatal nutrition, substance use, and stress (Figure 1). Certainly there are more epigenetic studies of these risk factors during the perinatal period than studies of PTB. Potential contributors to PTB influence gene regulation, are amenable to intervention, and have significant impacts on the future health of both the mother and child. Identification of biomarkers for these environmental risk factors would provide insight into the mechanisms underlying changes in DNA methylation that may be indicative of future health risk. Understanding how environmental risk factors affect DNA methylation would allow us to evaluate proposed biomarkers for PTB, separating out its clinical presentation from its risk factors. It should also be noted that many of environmental risk factors are related, a concept that is rarely addressed in studies that assess single risk factors in isolation. Thus, an epigenetic biomarker encompassing multiple indicators of the perinatal environment would be particularly valuable for reducing morbidity and mortality from PTB and related conditions. As of now, such a biomarker does not exist, but recent studies have made substantial progress towards this goal.

## 2. Preterm Birth

### 2.1. Health Burden of Preterm Birth

In 2013, 11.39% of neonates in the United States were born preterm (before 37 weeks gestation) [18]. PTB is the leading cause of infant mortality, accounting for ~35% of infant deaths, and contributes to disability among survivors [19]. Over the first week of life, a substantial fraction of deaths in neonates without congenital malformations result from PTB [20,21]. Children born preterm are more likely to have cerebral palsy, sensory deficits, learning disabilities, and respiratory illnesses [20,21]. PTB also increases the risk of being hospitalized with infections during childhood [22]. Among school aged children, those born preterm show diminished cognitive performance, increased externalizing and internalizing behaviors and are more likely to develop ADHD [23]. PTB and reduced fetal growth have also been linked to a number of important chronic diseases of adulthood such as type 2 diabetes [24,25]. Additionally, mothers who deliver preterm experience an increased risk of developing cardiovascular disease, type II diabetes, and breast cancer later in life [26,27,28,29]. Thus, PTB has substantial implications for the future health of both the mother and the neonate.

PTB is a heterogeneous condition, with three clinical types: medically indicated PTB, premature rupture of membranes (pPROM), and spontaneous preterm birth. Approximately 30%–35% of PTB is medically indicated due to a condition that places the mother or fetus at risk, including preeclampsia and fetal growth restriction [30,31]. pPROM accounts for another 25%–30% of PTB and occurs when the amniotic sac ruptures prematurely and more than an hour before the onset of contractions. [30]. Finally, spontaneous preterm birth refers to spontaneous labor and subsequent delivery before 37 weeks gestation, and accounts for between 23% and 64% of PTB. The underlying mechanisms of spontaneous PTB and pPROM are unknown [32]. Each type of PTB may have a unique biological signature, though there are likely to be common pathways as well because there are overlapping risk factors between the different types of PTB [33,34]. The idea that a single biomarker could be informative for different types of PTB is supported by a recent study examining DNA methylation in spontaneous PTB, pPROM, and medically indicated PTB that found no differences in eight candidate genes based on the type of PTB [35].

Mechanisms contributing to PTB have been recently reviewed in depth [36,37,38,39]. Briefly, spontaneous PTB has a range of contributing risk factors including infection, undernutrition, stress and substance use [39]. Two of the most common pathways linking these risk factors to preterm birth include: (1) activation of inflammatory and neuroendocrine pathways in response to stress, or stress-related behaviors such as smoking, resulting in upregulation of inflammatory cytokine production; and (2) activation of a cytokine-prostaglandin cascade in response to infection [21]. In this review, we will focus on spontaneous PTB where possible, though studies often do not distinguish between the different types of PTB or rely solely on gestational ages.

### 2.2. Epigenetics and Preterm Birth

The pathways underlying the associations between future neonatal development and future maternal disease risk with PTB remain unclear, but epigenetic dysregulation may contribute to a substantial part of this risk [36]. The most widely studied epigenetic change impacted by the environment is DNA methylation, a modification characterized by the addition of a methyl group to the 5’ position of a cytosine in a cytosine guanine dinucleotide (CpG) site. Typically, an increase in DNA methylation at promoter regions correlates with a decrease in expression of that gene, though exceptions to this are commonly documented [40]. Intragenic DNA methylation is also important to regulate alternative promoters and enhancers that define a variety of alternative transcripts and promote gene expression [41].

Microarrays and next generation sequencing have allowed examination of DNA methylation on a genome-wide scale. Due to this, several recent papers have examined the associations between the epigenome and PTB. Three studies have identified CpG sites related to PTB using neonatal blood [42,43,44]. Parets and colleagues found 29 CpG sites that were associated with spontaneous PTB independent of gestational age in a case-control study of 50 African American neonates. Several of these CpG sites were located in genes associated with development and the Notch signaling pathway. Additionally, 9637 CpG sites were associated with gestational age and were enriched among genes involved in embryonic development and the extra-cellular matrix [42]. The study by Cruickshank and colleagues identified 1555 CpG sites associated with PTB (no specific type defined) in 12 term and 12 preterm neonates, but did not adjust for gestational age. Interestingly, they found that most differences between term and preterm neonates observed at birth were no longer detectable at age 18 [43]. In a similarly powered study, Fernando and colleagues identified 1855 sites associated with spontaneous PTB from cord blood, 196 of which were independent of gestational age. These 196 sites were enriched for pathways associated with contractions and labor, but none of them overlapped with the 29 PTB-associated sites from the previous study of African American neonates. However, 157 differentially methylated sites overlapped with the Cruickshank analysis of PTB [44].

These studies support that there are DNA methylation differences in neonates born preterm, but there have been few studies examining methylation differences in mothers that deliver preterm. One recent study identified thousands of CpG sites in which neonatal methylation could be predicted from maternal methylation and that these correlated patterns were enriched among biological pathways implicated in PTB and chronic diseases [45]. These findings are supported by a recent study that examined maternal gene expression, which is often regulated by DNA methylation, related to preterm labor; 469 genes were differentially expressed in 106 women delivering preterm compared to 48 women with threatened preterm labor. In general, women with threatened preterm labor have persistent contractions, though only a small fraction will deliver within ten days. The genes identified were enriched for several pathways, including stress response and mRNA processing [46]. These findings suggest that there are substantial changes in gene regulation that can be detected in mothers prior to delivery in fetal or maternal tissues. Due to the current paucity of research in this area, future studies are needed to delineate the relationship between maternal gene regulation and PTB.

Although few studies have examined maternal blood for PTB-related epigenetic biomarkers, there are numerous studies examining the utility of other types of biomarkers in maternal serum such as cytokines and other metabolites [37,47,48,49,50,51,52,53]. One of the most widely reported serum biomarkers, alpha-fetoprotein, is currently used in prenatal screening and has been associated with increased risk of pregnancy complications, including PTB, as early as the second trimester [48,52,53]. These studies have shown promising associations. Because DNA methylation often regulates the expression of genes that encode proteins, biomarkers that assess methylation may allow for earlier identification of those at increased risk for delivering preterm. DNA methylation, unlike proteins, are also more amenable to screening with high-throughput panels that utilize standardized chemistry, and may allow for more rapid and reproducible assessment of multiple biomarkers. There have been several arguments for the use of methylation-based biomarkers for pregnancy complications, including preterm birth [54,55,56].

## 3. Developmental Origins of Health and Disease

Maternal behavior and environmental conditions can have a significant impact on the developing fetus through “fetal programming”. The Developmental Origins of Health and Disease (DOHaD) hypothesis was first proposed by David Barker after examining the association between birthweight and death rates in England and Wales [57]. The DOHaD hypothesis proposes that the uterine environment programs the fetus for environmental challenges that he or she is likely to experience after birth [57]. Thus, the uterine environment is crucial for long-term health and development, and identifying indicators of adverse uterine environments through the use of biomarkers may allow targeted interventions to change modifiable behaviors or mediate the negative effects of that environment.

A seminal example of prenatal programming affecting the exposed neonate after birth is the case of the Dutch Hunger Winter. Neonates exposed to this famine between 1944 and 1945 have experienced lasting effects on their health. [58,59,60,61,62]. One of the first studies on this cohort was published in 1976, when the exposed neonates would have been 19 years of age. This study by Ravelli and colleagues revealed that the timing of famine exposure greatly impacted future health, with men exposed to famine in the first and second trimesters more likely to be obese at age 19, and men exposed to famine during the third trimester experienced obesity at a much lower rate through age 19 [58]. Since 1976, a plethora of follow-up studies have been published on this cohort. In addition to the findings of Ravelli and colleagues, subsequent studies have seen associations between famine exposure and PTB, low birth weight (LBW), neural defects, schizophrenia spectrum disorders, addiction in men, impaired glucose tolerance, and earlier occurrence of menopause [59,60,61,62].

Recent studies, including several presented in this review, have explored the plausibility of developmental programming taking place through epigenetic changes associated with the prenatal environment [63]. The myriad of health consequences associated with prenatal exposure to famine provides evidence in support of the DOHaD hypothesis. For example, severe undernutrition, such as in the case of the Dutch Hunger Winter, has also been associated with changes in methylation, in addition to its associations with weight, neurological and endocrine consequences [64]. Tobi and colleagues examined methylation at 15 candidate loci in this population. They found changes in methylation at several imprinted and non-imprinted genes with functions related to metabolic and cardiovascular disease as compared to siblings not exposed to famine [64]. Imprinted genes provide a unique opportunity to measures changes in DNA methylation as silencing of the imprinted allele occurs early in development and is stably transmitted [65]. Thus, when one allele in a diploid cell is imprinted, the differentially methylated regions (DMRs) should exhibit 50% methylation under normal circumstances, allowing changes in methylation due to the intrauterine environment to be readily detected [65]. These results indicate that famine during the prenatal period can have lasting effects on the epigenome of the fetus, and future studies should evaluate the methylation of these genes in undernourished populations. The DOHaD hypothesis is further illustrated in a recent study by Silver and colleagues [66]. They found that the methylation at a metastable epiallele, vault RNA 2-1 (*VTRNA2-1*), was associated with season of conception and maternal nutrition in rural Gambia, suggesting that the early prenatal environment can “program” fetal methylation that is stable long-term [66]. Future studies identifying other stable methylation changes associated with the periconceptional environment and factors contributing to preterm birth are needed and may provide candidate biomarkers predictive of the intrauterine environment and long-term health risks through adulthood.

## 4. Nutrition

### 4.1. Maternal BMI

Maternal over and undernutrition can have significant impacts on pregnancy outcome for both the mother and fetus [67,68,69,70,71,72,73]. The primary maternal outcome associated with low maternal body mass index (BMI) was delivering preterm [67]. The risk of delivering preterm was associated with the degree of maternal undernutrition, but this association could be mitigated by appropriate weight gain during pregnancy [67]. Low maternal BMI is also associated with fetal growth restriction [67,68,69].

Sharp and colleagues performed an epigenome-wide association study (EWAS) for associations between DNA methylation and maternal BMI in the Avon Longitudinal Study of Parents and Children cohort. They found 1621 CpG sites associated with low maternal BMI in offspring, though no sites were associated with gestational weight gain [9]. When examining long-term offspring adiposity in childhood and adolescence, hypomethylated sites tended to be positively associated with adiposity in children born to underweight mothers and negatively associated with adiposity in children born to overweight mothers. Hypermethylated sites tended to be negatively associated with adiposity in children born to underweight mothers and positively associated in children born to overweight mothers. However, only one CpG site was differentially methylated based on adiposity at both birth and adolescence.

The rate of PTB in obese women is lower than in the general population [68]. However, high maternal BMI is also associated with preeclampsia and ensuing preterm delivery, gestational diabetes, and hypertension. High maternal BMI also associated with increased risk of cesarean section, prolonged hospital stay, and postpartum hemorrhage in the mother and increased risk of stillbirth, macrosomy, low Apgar score, meconium aspiration, seizures and cerebral palsy in the neonate. [68,69,73,74,75,76]. In an analysis of population attributable risk fractions, Oteng-Ntim and colleagues found that eliminating or reducing maternal obesity could circumvent a significant proportion of negative outcomes [77].

Numerous studies have examined the link between overweight and obese mothers and offspring DNA methylation [78,79,80]. Nomura and colleagues found that maternal obesity is associated with decreased global methylation in the placenta, but not in umbilical cord blood, raising the question of which tissues should be interrogated when examining the association between maternal obesity and fetal methylation, though the issue of tissue specificity is broadly relevant to all EWAS studies [78]. They also saw an association between placental hypermethylation and two neonatal outcomes: smaller head circumference and shorter body length [78]. However, another recent study did not see an association between global methylation and maternal BMI in either the placenta or cord blood [79].

Liu and colleagues examined DNA methylation and maternal BMI in a cohort of African American women and found that male and female neonates displayed distinct associations at different CpG sites, though none remained significant after multiple test correction [80]. Even so, they raise the point that many of the CpG sites approaching significance for association between maternal BMI and child methylation have previously been implicated in a variety of biological pathways significant in the development of cancer and other chronic diseases [80].

Maternal obesity may influence offspring outcome by altering the immune system of both the mother and the fetus [70,72,81,82,83,84]. Obesity is associated with increased cytokine production, and may alter nutrient transport and placental vasculature [70,72,81,82]. Cytokine exposure may also alter gene expression or epigenetic marks in the fetus, eventually leading to altered brain development and increased risk for obesity, though the mechanistic link between cytokines and these outcomes is unclear [81].

Weight loss has profound effects for the health of the mother and the fetus, as seen through studies on the effects of gastric bypass surgery [85,86]. In addition to weight loss, gastric bypass surgery has been associated with reduction in rates of type II diabetes, hypertension, and hyperlipidemia, and these comorbidities are associated with pregnancy complications for the mother and fetus [87,88,89]. Gastrointestinal bypass surgery provides an opportunity to examine methylation difference in siblings conceived before and after surgery [86]. Offspring born after maternal surgery are less obese and have better cardiovascular risk profiles than their siblings born before surgery [86]. There is also a significant difference in the methylation profiles of siblings born before and after surgery, after adjusting for age, with 14,466 CpG sites in 5698 genes being differentially methylated between sibling groups. The 5698 genes were enriched in pathways related to autoimmune, pancreas, and glucose metabolism disorder [86]. This implies that maternal weight loss may have enormous implications for the methylome of her offspring, as well as their future health. CpG sites associated with weight loss should be examined for associations with the offspring outcomes in mothers not undergoing surgery to identify whether methylation at these CpG sites mediates the relationship between maternal weight, child obesity and cardiovascular risk profiles. If these CpG sites are found to mediate the relationship, they may serve as potential epigenetic biomarkers to identify and monitor children at risk for childhood obesity and cardiovascular risk factors so that preventive steps may be taken.

### 4.2. Folate 

Folate is recommended during pregnancy to prevent neural tube defects [90,91]. Folate deficiency is associated with maternal depression, bacterial vaginosis, and preeclampsia, all of which increase risk for PTB, although the association between folate levels and preeclampsia is controversial [92,93,94,95,96]. Folate supplementation has also been shown to positively impact the fetus by decreasing rates of PTB as well as being born with low birth weight and small for gestational age [92,93,94,95,97,98,99]. Despite the success of folate supplementation in reducing neural tube defects, it may be associated with other fetal complications including cleft lip/alveolus and bronchiolitis [100,101].

The role of folate intake during pregnancy and its impact on offspring outcome and epigenetics has been widely studied [102,103,104,105,106]. Folate is a methyl donor, and methyl donor deficiency correlates with changes in DNA methylation [102,103,104,105,106]. Folate supplementation use after 12 weeks gestation has been associated with differences in global methylation (LINE1) and methylation patterns of imprinted genes (*IGF2*, *PEG3*) [107,108,109]. These associations are present in both in maternal and umbilical cord blood [107]. More research into the potential correlations between altered methylation at these sites and neonatal outcomes is needed. It is possible that hypomethylation of LINE1 elements could lead to genomic instability by mobilizing transposable elements. Similarly, aberrant methylation of imprinted regions has previously been associated with cancer, infertility, and transient neonatal diabetes, among others [107,110,111,112].

Hoyo and colleagues did not see a relationship between folate supplementation in *IGF2*, but *H19* methylation was significantly decreased with folate supplementation in male neonates [103]. There was no association with folate supplementation in females [103]. Ba and colleagues also saw no changes in *IGF2* methylation associated with folate supplementation, although methylation was impacted by levels of vitamin B12 [113]. Despite these discordant findings, examining differential methylation in genes known to associate with folate levels may be useful for personalizing folate doses to better balance the risk associated with too much or too little folate intake.

### 4.3. Vitamin D

Vitamin D deficiency in pregnancy has been associated with increased incidence of PTB, small for gestational age, and low birth weight in neonates [114,115,116,117,118]. A recent meta-analysis did not see a positive change in PTB, LBW, pre-eclampsia, and gestational diabetes rates with vitamin D supplementation, though they did conclude that vitamin D has “plausible effects” on many perinatal outcomes [119]. Maternal vitamin D levels during pregnancy also predict long-term outcomes for the child including congenital rickets, smaller arm muscle and higher insulin resistance, autism spectrum disorders, and asthma in childhood. Higher levels of vitamin D is associated with higher fasting insulin, cholesterol, and triglyceride concentrations in adults [120,121,122,123,124].

The largest predictor of circulating vitamin D levels in neonates is the circulating vitamin D levels of their mothers [125]. Circulating vitamin D is converted to 1,25-dihydroxyvitamin D (25-OHD), and can be further converted into its hormonal form, 1,25(OH)2D. This is typically accomplished in the kidneys, but the placenta is also capable of synthesizing 1,25(OH)2D [126]. 1,25(OH)2D then binds the vitamin D receptor, which can subsequently act as a transcription factor by targeting the vitamin D response element (VDRE) motif within the promoter of the target gene to affect gene expression [126].

Vitamin D Binding Protein (VDBP) has recently been assessed as a biomarker of preterm labor, and shows promise as a potential predictor [127,128]. Liong and colleagues found that cervicovaginal fluid concentrations of VDBP were elevated in the days preceding labor relative to the rest of pregnancy [127]. Women who delivered preterm were also found to have higher baseline levels of VDBP. They concluded that VDBP levels had high positive and negative predictive values (82.8% and 95.3%) [127]. In a subsequent study, when combining VDBP and albumin levels, the positive and negative predictive values increased (100% and 96.7%) [128].

The effects of maternal vitamin D levels may influence perinatal and long-term outcomes through immune, antibacterial, and anti-inflammatory responses [99,129]. A recent study has found associations between vitamin D levels in maternal and cord blood and immune cell type proportions, as well as placental expression of immune system related genes [130]. Interestingly, vitamin D deficiency was also associated with downregulation of the expression of vitamin D receptor (VDR), *FOXP3* (a transcription factor associated with Treg cells), and retinoic acid receptor (RAR). It was also associated with upregulation of the expression of *VDRB*, complement receptors *CD21* and *CD23,* and a Vitamin D regulatory enzyme (*CYP24A1*) in the placenta [130].

Of note, 25-OH-D concentrations may themselves serve as a biomarker for negative outcomes, especially preterm birth and preeclampsia, although these associations are controversial [119,131,132,133]. Accumulating data support that vitamin D deficiency plays a role in disparities in reproductive health outcomes [132,134]. A recent study reported that methylation of a subset of CpG sites identified through a network analysis is affected by both ancestry and maternal vitamin D concentrations, suggesting that both of these factors may impact the methylome of the neonate [135].

## 5. Substance Use

### 5.1. Maternal Smoking during Pregnancy

Smoking is a well-known, preventable, significant contributor to neonatal and maternal morbidity and mortality, yet 12.3% of pregnant women smoked in 2010 [136]. Smoking may cause complications for the fetus due to exposure to tobacco toxins, poor umbilical blood flow, oxidative stress, and changes in gene expression [137,138,139]. Conditions associated with maternal smoking in pregnancy for the neonate include PTB, fetal growth restriction, sudden infant death syndrome, stillbirth, paraventricular leukomalacia, bronchopulmonary dysplasia, intraventricular hemorrhage, placenta-associated syndrome as well as reduced academic performance and elevated blood pressure in adolescence [3,136,137,140,141,142,143,144,145].

In adults, DNA methylation differences in the blood of smokers *versus* non-smokers have been reported [10,146]. Maternal smoking during pregnancy may change the DNA methylation profile of neonates, as evidenced in several recent studies, and this change in methylation may mediate neonatal birthweight and immune function [13,147,148,149]. Stroud and colleagues found that maternal smoking during pregnancy was associated with decreased infant salivary cortisol levels in the first post-natal month, and that placental *NR3C1* methylation was decreased in exposed fetuses [13]. They suggest that decreased placental methylation of the *NR3C1* promoter mediates the lowered cortisol levels seen in neonates [13]. Other groups have found associations between maternal smoking in pregnancy and methylation through EWAS [147,148,149]. The association between maternal smoking and methylation in the offspring can be seen through adolescence, suggesting that there is the potential for long-term effects of this behavior on the child [150,151]. One group, Kupers and colleagues, performing an EWAS with methylation and maternal smoking was able to establish the mediating effect of three CpG sites associated with growth factor independent 1 transcription repressor (*GFI1*) on low birth weight, concluding that these CpGs could explain 12%–19% of the lowered birth weight [149]. Future studies should take a similar approach when evaluating the effects of maternal exposures on fetal and neonatal outcome.

### 5.2. Alcohol

Alcohol use during pregnancy is reported by around 15% of women and is another preventable contributor to maternal and neonatal morbidity and mortality [152]. Alcohol use during pregnancy is associated with PTB, spontaneous abortion, fetal alcohol spectrum disorder, increased risk of acute myeloblastic leukemia, and poor childhood behavior [152,153,154,155]. Kesmodel and colleagues found that prenatal alcohol exposure decreased gestational age by 3–4 days, on average, in women consuming more than 10 drinks per week, but gestational age was increased by 1–2 days in women having 1–2 drinks per week [156]. Lundsberg and colleagues also saw a protective effect of alcohol use in PTB, though this phenomenon may be due to a “lifestyle effect” where women who consume low or moderate levels of alcohol may participate in an overall healthier lifestyle [157]. In adults, alcohol use has also been shown to affect the epigenomes of recurrent users, with particular implications for dysregulation of inflammatory pathways [158,159].

Changes in methylation level of specific genes may mediate the effects of alcohol exposure on fetal outcomes, though few studies have examined this relationship. Lee and colleagues found a variety of associations between maternal alcohol consumption and cord blood methylation in the fetus [160]. In this study, serotonin transporter (*SLC6A4*) methylation was decreased and methyl CpG binding protein 2 (*MECP2*) methylation was increased in maternally exposed newborns, and as both of these genes have previously been implicated in neurodevelopment, altered methylation may contribute to negative neurocognitive outcomes in prenatally exposed children [160]. Future studies should evaluate the association between CpG sites associated with alcohol use in the mother and PTB to better assess potential biomarkers of PTB for use in subpopulations of women who use alcohol during pregnancy. Biomarkers of maternal alcohol use in the neonate that are predictive of future neurocognitive function should be developed to allow earlier intervention and diagnosis to potentially improve long-term outcomes for at risk neonates.

## 6. Prenatal Stress

Maternal stress during pregnancy is an overwhelmingly common event, with 70.2% of women reporting the occurrence a stressful life event in the year preceding the birth of their child including pregnancy-specific stress, intimate partner violence, and natural disasters [161]. The number of stressful life events was highest for women at high risk for pregnancy complications (*i.e.*, under 25 years of age, less than a high school education, African American race, unmarried, or receiving Medicaid) [161].

Maternal stress has been associated with lower gestational age, even in cases not strictly defined as PTB. Lobel and colleagues report that maternal pregnancy specific stress was predictive of gestational age, with women who had a combination of high obstetric risk and high stress delivering, on average, nine days earlier than their low stressed/low risk counterparts [162]. They note that the association between pregnancy specific stress and gestational age was still significant after controlling for obstetric risk, but poor health behaviors may also mediate the association [162].

The effects of maternal stress can be seen through dysregulation of the hypothalamic-pituitary-adrenal (HPA) axis and activation of inflammatory immune pathways [36]. Stress promotes release of corticotropin-releasing factor (CRF), which stimulates release of adrenocorticotrophin (ACTH) into circulation [163]. ACTH then stimulates the synthesis and secretion of glucocorticoids [163], which then feedback to inhibit further release of CRH and ACTH [163,164]. Cortisol binds the glucocorticoid receptor (*NR3C1*), which can then translocate into the nucleus to either bind glucocorticoid response elements on their target genes or interact with other transcription factors, such as NF-κB [164]. Immunosuppression via glucocorticoids has been associated with decreases in cytokine production and function, impaired leukocyte access to inflamed regions, and reduction of the inflammatory response [165,166].

The levels of CRH, ACTH, cortisol, and cortisol binding protein (CBP) all increase over the course of pregnancy [167]. The placenta is also capable of synthesizing CRH to supplement CRH synthesized by the hypothalamus [168]. Increased CRH induces increased secretion of ACTH and cortisol, contributing to a positive feedback loop [5]. The neonate is protected from rising levels of cortisol by the actions of 11-β hydroxysteroid dehydrogenase type 2 (HSD11B2), which converts cortisol into cortisone [5,169]. However, very high levels of maternal cortisol, like those seen during periods of maternal stress, may override the protective actions of this enzyme, which provides a potential link between methylation of *HSD11B2* and the intrauterine environment [169].

Vidal and colleagues attempted to identify just such a biomarker by examining the relationship between perceived maternal stress, measured on the Perceived Stress Scale (PSS), and neonatal methylation at imprint regulatory regions [170]. The PSS was developed as a method of measuring an individual’s perception of a stressful event based on their personal interpretation of the circumstances through 14 questions answered on a numerical gradient corresponding to experiencing a certain emotion “never” to “very often” [171]. Vidal and colleagues found one differentially methylated region in cord blood that associated with PSS: the Mesoderm Specific Transcript (*MEST*) DMR [170]. *MEST* has been associated with placental mesenchymal dysplasia, which may indicate the presence of other underlying conditions [172].

Natural disasters provide a unique perspective on stress during pregnancy because they affect women agnostically, regardless of race, socioeconomic status, and other dividing characteristics. The first example of natural disaster related stress is from Project Ice Storm, which followed up with women who were pregnant around the time of the Quebec ice storm [173]. This study examined the methylation profiles of 36 offspring willing to provide blood samples around thirteen years of age. They found 1675 CpG sites were associated with objective maternal stress levels, though no sites were significantly associated with subjective maternal stress [173]. In this study, subjective maternal stress was measured by the Impact of Events Scale-Revised, which assesses emotional and psychological symptoms after a traumatic exposure, while objective stress was measured by a questionnaire assessing distinct and measurable hardships, like damage to residence [173,174]. The results of this study bolster the hypothesis that methylation changes induced before birth can still be seen into adolescence.

A second natural disaster case study highlights the effects of socioeconomic status on stress. Following Hurricane Andrew that struck Louisiana in 1992, there was a significant increase in the rate of preterm birth in affected areas, regardless of race [4]. However, PTB was increased only in African American populations living in unaffected areas after controlling for race. The authors suggest that portions of the population already experiencing high levels of stress and health disparity may be disproportionately affected by acute stress, and further research is needed to identify the epigenetic and biological pathways behind this phenomenon [4].

In addition to acute stress, chronic stress also contributes to an adverse intrauterine environment for the fetus. There has been considerable debate in the field as to the effects of chronic stress on cortisol levels [175]. However, chronic stress is more likely to produce the types of epigenetic changes that may be informative as a biomarker. One potential source of chronic stress is intimate partner violence (IPV), which is associated with adverse maternal pregnancy outcomes in pregnancy, including delivering preterm, hemorrhage, preeclampsia, vaginal bleeding, hypertension, uterine rupture, spontaneous abortion, and maternal death [6,176]. In a recent meta-analysis, domestic abuse was associated with both PTB and LBW [177].

The effects of intimate partner violence can be perpetuated epigenetically in the fetus, and are still evident into adolescence [178]. When examining methylation of the glucocorticoid receptor gene, Radtke and colleagues saw a significant association between methylation and maternal IPV experience during pregnancy. However, IPV preceding or following pregnancy had no effect on *NR3C1* methylation of adolescents [178]. This finding supports the hypothesis that maternal stress in pregnancy influences the HPA axis in her offspring, and these effects can be seen into adolescence, and possibly beyond. Thus, the *NR3C1* gene should be further investigated for associations with adverse outcomes in the fetus and later in life. A biomarker for maternal stress in pregnancy could allow identification of at risk women so that support systems to diffuse pregnancy-specific stress could be put into place [162].

### Support during Pregnancy

Support during pregnancy may ameliorate some of the adverse maternal and neonates outcomes associated with maternal prenatal stress [179,180,181,182]. In an Ethiopian cohort, social support was associated with a lower likelihood of reporting depressive symptoms in pregnancy [179]. The association between depressive symptoms and support from a pregnant adolescent’s mother and/or partner was also seen by Pires and colleagues in a Portuguese cohort [181]. These studies suggest that maternal support during pregnancy can reduce depressive symptoms experienced by the mother. This is significant, as depression is a risk factor for PTB, and identifying women at risk for depression during pregnancy could allow for the implementation of support systems and treatment to improve maternal quality of life and decrease the PTB rate.

Support in pregnancy can also positively influence child outcomes. Ghosh and colleagues found that partner support during pregnancy was associated with lower rates of PTB in a primarily Latina population residing in Los Angeles [180]. In this study, women under moderate to high levels of stress were at a higher risk of delivering preterm, but partner support did mediate this effect. They also note that African Americans reported the highest levels of chronic stress [180]. However, Straughen and colleagues were unable to replicate the mediating effects of social support on PTB in African Americans [182]. Giesbrecht and colleagues assessed the influence of support in pregnancy on the HPA axis of the mother through evaluating cortisol levels. They found that social support was able to mediate the effects of stress on cortisol levels, as women with effective social support showed only modest increased in cortisol in response to stress as compared to women who did not perceive their partners as supportive [183]. Therefore, support during pregnancy may protect the fetus from exposure to high cortisol levels in utero [183].

Several strategies for reducing stress and providing support during pregnancy have been evaluated, including yoga, massage therapy, massage therapy by a partner, and group prenatal care [184,185,186,187,188,189,190]. These studies have shown that the negative effects of stress during pregnancy may be mediated by certain interventions [184,185,186,187,191]. These interventions have measurable effects on levels of serotonin, dopamine, and cortisol, which could be an indicator of successful intervention [191]. Massage therapy, especially moderate pressure massage, has also shown benefits for the newborn, as assessed by their performance measured on the Brazelton Neonatal Behavioral Assessment [186,191]. Group prenatal care revolves around a model in which pregnant women engage in a small group with other women due at the same time, and with their provider at prenatal visits. This model has been shown to increase gestational length and birth weight, though reports of these benefits have been inconsistent [188,189,190]. Group prenatal care has also been shown to have significant benefits for maternal psychosocial outcomes, including reducing pregnancy-specific distress and post-partum depression, in women with low social support [192]. Combinations of multiple support systems could show greater benefits. To our knowledge, previous studies have not examined the effects of these interventions on the methylomes of either mother or child. Massage therapy by a partner is a cost-effective intervention for maternal stress, and its effects on biological indicators of maternal and neonatal health should be examined.

## 7. Health Disparities

African Americans experience PTB at a rate nearly 1.5-fold that of Caucasians [18]. Differences in known risk factors, such as socioeconomic status, fail to explain this disparity [193,194]. The excess risk in African Americans may be due to a number of underlying factors including increased activation of inflammatory pathways, exposure to stress, poor nutritional status, and an increased rate of reproductive tract infections [21,195,196,197]. They may also disproportionately experience health-related ramifications in response to stressful life events, as illustrated by the study of Hurricane Andrew in which only African American women had higher rates of preterm delivery [4]. Genetic and epigenetic factors have also been proposed to explain the disproportionate PTB risk among African Americans. Candidate gene studies have identified polymorphisms associated with PTB in African American cohorts, but many have failed to replicate and do not explain a significant proportion of the racial disparity [31,198]. Two recent studies have identified race-specific methylation differences that are identifiable at birth [135,199]. Still, indicators of socioeconomic status are also evident at birth, as Tehranifar and colleagues found that low socioeconomic status associated with higher methylation of a repetitive element, *Sat2* [200]. Another study found gene-specific differences in stress-response pathways related to early life socioeconomic status [201]. It is likely that each of these factors increases risk for PTB incrementally without explaining a substantial proportion of the variance on its own.

### Maternal Weathering

The maternal weathering hypothesis, first proposed by Geronimus, postulates that the growing disparity with maternal age in pregnancy complications may be reflective of accelerated aging due to disproportionately experienced stressors [202,203,204]. Stress may cause accelerated aging through oxidative stress, telomere shortening, or acceleration of the “epigenetic clock”, discussed below [205,206,207]. In this context, accelerated aging refers to an increase in cellular age over chronological age. This hypothesis has been supported through the demonstration of a divergence in risk for PTB between African American and Caucasian cohorts that were otherwise comparable [204]. Thus, age acceleration may be associated with pregnancy complications in women with disproportionately higher levels of stress.

The original weathering hypothesis was focused on differences in pregnancy complication rates between African Americans and Caucasians, though it has recently been tested in Mexican-American and Caucasian populations. However, there is little evidence for weathering in these populations, suggesting that wreathing may be specific to African Americans and not generalizable to members of other racial groups with low socioeconomic status [204,208,209,210]. Further examination of accelerated aging is required to better understand its potential contributions to preterm birth.

## 8. Accelerated Aging

### 8.1. Telomere Length and Cellular Senescence

Accelerated aging, as measured through telomere length, has recently been interrogated for associations with PTB [211,212,213,214]. Telomeres cap the ends of chromosomes to promote chromosomal stability and aid in replication. The length of telomeres, a hallmark of aging, is regulated by the enzyme telomerase, which adds additional nucleotides to the chromosome ends to help correct for telomere shortening that occurs with each cell division [215,216]. Previous studies have shown that telomere length associates with PTB, pPROM, still birth, preeclampsia, and intrauterine growth restriction, though the mechanisms driving these associations remain to be elucidated [211,212,217,218,219]. Menon and colleagues reported an inverse relationship between cord blood leukocyte telomere length and gestational age, noting that leukocytes from neonates born preterm had longer telomeres than leukocytes form neonates born either at term or with pPROM [212]. This finding is in contrast with Friedrich and colleagues, who reported no difference between telomere length in cord blood leukocytes of term and preterm neonates, although they did see a difference in telomere length between very low birth weight preterm neonates and low birth weight preterm neonates [213]. This discrepancy may be due to differences in the way telomere length was measured in these the two studies. Friedrich and colleagues also reported a decrease in telomere length between 27 and 32 weeks gestation, consistent with the findings from Menon and colleagues, but did not see an association with telomere length after 33 weeks [213]. Menon and colleagues also saw a strong correlation between telomere length in leukocytes and placenta, though placental telomeres were longer than those of leukocytes on average [212].

In a more recent study, Ferrari, Menon, and colleagues examined telomere length in placentas from 42 stillbirths, 43 term, and 15 preterm live births. They found that, overall, placental telomere length was two-fold shorter in placentas from stillbirths. Interestingly, they also note that telomere length in pPROM was similar to telomere length in stillbirths. These results suggest that premature senescence and placental accelerated aging are associated with the severe negative outcomes of stillbirth and pPROM [211]. Both of these studies, in addition to the findings of Biron-Shental and colleagues, which suggests that pre-eclampsia and intrauterine growth restriction are associated with shorter telomeres, imply that telomere length may be used as a proxy for cellular aging and senescence [211,212,219].

Consistent with the hypothesis that telomere length associates with cellular aging and senescence, shortened telomeres have been associated with a variety of negative health outcomes including cancer, dementia, stroke, type II diabetes, myocardial infarction, psychiatric disorders, and mortality, though direct causal relationships have not been established [220,221,222,223,224,225]. Therefore, beginning life with shorter telomere length may predispose a neonate to long-term health consequences. These results are consistent with a study by Shalev and colleagues that found perinatal complications associated with shorter telomeres and older perceived facial age at age 38 [226].

In addition to associations with pregnancy complications and overall health, the impact of maternal stress on telomere length in neonates has been studied. Entringer and colleagues examined telomere length in adult subjects exposed to maternal stress during pregnancy and found that stress exposure associated with shorter telomere length in adults, and this effect was more pronounced in female offspring exposed to stress [227]. Although this study did not examine the direct relationship between telomere length at birth and maternal stress, a more recent study by Entringer and colleagues found that pregnancy-specific stress did associate with newborn telomere length in a cohort of 27 mothers and neonates [228]. Due to these vast associations between telomere length, PTB, pregnancy complications and general poor health, telomere length is attractive as potential biomarker of future neonatal development.

### 8.2. Epigenetic Clock

Recently, DNA methylation of a select group of CpG sites has been used to generate a promising new biomarker related to aging. It is widely accepted that aging influences DNA methylation across the genome, and several recent studies have taken advantage of age-related methylation changes to build a predictor of DNA methylation age (DNAm age) [229,230,231]. A DNAm age that is higher than a person’s chronological age may indicate accelerated aging, which is a potential metric of stress, as evidenced by recent studies of DNAm age in developmental and neurocognitive outcomes, as well as all-cause mortality [207,232]. Measuring DNAm age has potential for use in predicting maternal risk during pregnancy, though this relationship has not yet been examined. One study has examined the relationship between accelerated age and a range of phenotypes in a large, longitudinal cohort. Overall, they found that DNAm age became more correlated as the child aged, suggesting that this predictor may not work well for neonates and young children. Accelerated age measured at birth associated with Caesarian-section and maternal smoking (*p* < 0.05), though accelerated age measured in childhood and/or adolescence associated with birthweight and maternal characteristics measured during pregnancy including selenium exposure and BMI [233].

Placental and cord blood samples were included when the DNAm age predictor was initially developed, but the age of all of these samples was set to 0. Due to this, DNAm age, as initially operationalized, is not accurate enough to discriminate weeks of gestation. Gestational age has previously been associated with changes in methylation at various CpG sites [11,42,234,235], which could be used to develop a predictor accurate for neonates. Further studies on DNA methylation in neonatal cord blood are required for the development of a neonatal age predictor.

## 9. Conclusions and Recommendations

**Figure 2:**
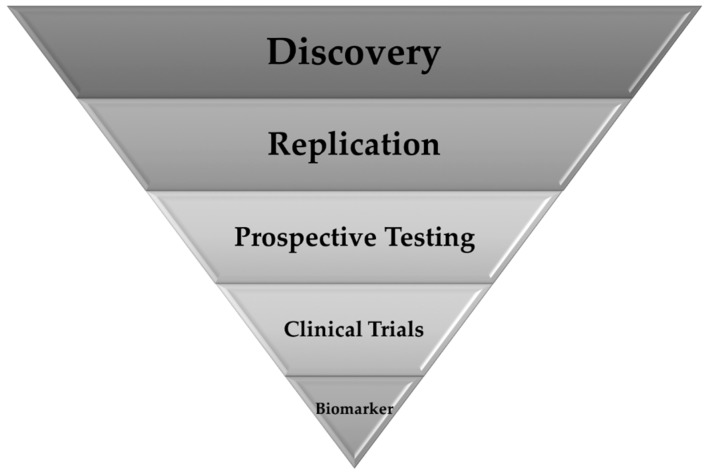
Phases of biomarker development. The width of each phase is analogous to the number of studies anticipated.

Figure 2 summarizes the process of identifying an epigenetic biomarker and underscores the complexity of developing one for PTB or its risk factors. While the clinical need is clear, an epigenetic biomarker for PTB would substantially improve our ability to identify and treat women at the highest risk for delivering preterm. Targeted interventions could reduce multiple health burdens associated with delivering preterm and being born preterm. Such a biomarker does not yet exist, in part due to the complexity of the intrauterine environment and the heterogeneity within the clinical definition of PTB. Gene regulation varies over the course of pregnancy and that regulation is likely influenced by a number of independent environmental factors. Thus, potential biomarkers would need to be capable of distinguishing health outcomes within diverse nutritional or psychosocial contexts. Studies that simultaneously evaluate multiple risk factors for PTB will have the most potential to identify epigenetic predictors of PTB that can be developed into biomarkers. Similarly, biomarkers should perform equally well across different ancestries and socioeconomic statuses, as recent studies have identified methylation differences in ancestry and SES that are evident at birth [135,199,200]. Recent studies have made substantial progress towards identifying and replicating epigenetic associations in diverse cohorts, and it is important that basic and epidemiological researchers develop partnerships though which the most promising results from their work can be extended into prospective and clinical studies.

In this review, we have outlined current progress on biomarker development and the need for additional epigenetic studies of PTB. In addition to individual CpG sites, summary measures that integrate information from multiple regions of the genome may also be informative. The use of the DNAm age predictor to evaluate future health risk in adults demonstrates that this type of epigenetic predictor, which combines several hundred CpG sites, may be useful for predicting perinatal development, if modified for use in neonates. Once appropriate biomarkers are identified, targeted and cost-effective assays could be developed to prospectively screen for PTB. Prospective screening is an essential stage in development of biomarkers for the assessment of whether statistical associations between PTB and DNA methylation will have clinical utility and be useful for accurate and rapid prediction of PTB. Successful development and clinical implementation of such a biomarker would aid in reducing the substantial health burden of PTB and has the potential to improve both maternal and offspring health.

## Abbreviations

The following abbreviations are used in this manuscript:

25-OHD	1,25-dihydroxyvitamin D
ACTH	adrenocorticotrophin
AED	Antiepileptic drugs
BMI	Body Mass Index
CBP	Cortisol binding protein
CpG	Cytosine Guanine dinucleotide
CRH	Corticotrophin-releasing hormone
DMR	Differentially methylated region
DNAm age	DNA methylation age
EWAS	Epigenome-wide association study
GFI1	Growth factor independent 1 transcription repressor
HPA axis	Hypothalamic-pituitary-adrenal axis
*HSD11B2*	Hydroxysteroid dehydrogenase type 2
*IGF2*	Insulin-like growth factor 2
IPV	Intimate partner violence
LBW	Low birth weight
MECP2	Methyl CpG binding protein 2
*MEST*	Mesoderm Specific Transcript
miRNA	microRNA
*NR3C1*	Glucocorticoid receptor
*PEG3*	Paternally Expressed 3
pPROM	Preterm, premature rupture of membranes
PSS	Perceived stress scale
PTB	Preterm birth
RAR	Retinoic acid receptor
SES	Socioeconomic status
VDBP	Vitamin D Binding Protein
VDR	Vitamin D Receptor
VTRNA2-1	Vault RNA 2-1
